# Prenatal ethanol exposure and changes in fetal neuroendocrine metabolic programming

**DOI:** 10.1186/s40659-023-00473-y

**Published:** 2023-11-17

**Authors:** Liang Liu, Yinxian Wen, Qubo Ni, Liaobin Chen, Hui Wang

**Affiliations:** 1 Department of Orthopedic Surgery, Joint Disease Research Center of Wuhan University, Zhongnan Hospital of Wuhan University, Wuhan, 430071 China; 2 Hubei Provincial Key Laboratory of Developmentally Originated Disease, Wuhan, 430071 China; 3 Department of Pharmacology, Wuhan University School of Basic Medical Sciences, Wuhan, 430071 China

**Keywords:** Intrauterine programming, Neuroendocrine metabolism, Prenatal ethanol exposure, Glucocorticoid, Developmental origins of Health and Disease (DOHaD)

## Abstract

Prenatal ethanol exposure (PEE) (mainly through maternal alcohol consumption) has become widespread. However, studies suggest that it can cause intrauterine growth retardation (IUGR) and multi-organ developmental toxicity in offspring, and susceptibility to various chronic diseases (such as neuropsychiatric diseases, metabolic syndrome, and related diseases) in adults. Through ethanol’s direct effects and its indirect effects mediated by maternal-derived glucocorticoids, PEE alters epigenetic modifications and organ developmental programming during fetal development, which damages the offspring health and increases susceptibility to various chronic diseases after birth. Ethanol directly leads to the developmental toxicity of multiple tissues and organs in many ways. Regarding maternal-derived glucocorticoid-mediated IUGR, developmental programming, and susceptibility to multiple conditions after birth, ethanol induces programmed changes in the neuroendocrine axes of offspring, such as the hypothalamus-pituitary-adrenal (HPA) and glucocorticoid-insulin-like growth factor 1 (GC-IGF1) axes. In addition, the differences in ethanol metabolic enzymes, placental glucocorticoid barrier function, and the sensitivity to glucocorticoids in various tissues and organs mediate the severity and sex differences in the developmental toxicity of ethanol exposure during pregnancy. Offspring exposed to ethanol during pregnancy have a “thrifty phenotype” in the fetal period, and show “catch-up growth” in the case of abundant nutrition after birth; when encountering adverse environments, these offspring are more likely to develop diseases. Here, we review the developmental toxicity, functional alterations in multiple organs, and neuroendocrine metabolic programming mechanisms induced by PEE based on our research and that of other investigators. This should provide new perspectives for the effective prevention and treatment of ethanol developmental toxicity and the early prevention of related fetal-originated diseases.

## Introduction

Drinking can be harmful to individuals [1–4]. Epidemiological investigation shows that an estimated 10% of pregnant women consume alcohol globally, and prenatal ethanol exposure (PEE) has further increased in recent years [5, 6]. Many studies have shown that PEE has a more obvious damage to the fetus than the mother due to immature development and incompletely established homeostasis, which not only induces fetal developmental toxicity (including intrauterine growth retardation (IUGR) and fetal alcohol syndrome (FAS)), but also can lead to a series of long-term development-related health problems and susceptibility to conditions in adulthood (such as neuropsychiatric disorders and metabolic syndrome (MS)) [1–4, 7–9]. In the 1990s, Dr. David Barker performed a large-scale epidemiological investigation and found that the incidence of coronary heart disease, hypertension, hyperlipidemia and obesity was increased in adults with IUGR; he thus proposed the hypothesis of the intrauterine origin of adult diseases [10–13]. Over the past three decades, numerous scholars have continued studies on the relationship between adverse environments during pregnancy, birth weight and the development of diseases in adults, and put forward a new concept — the Developmental Origins of Health and Disease (DOHaD). Upon further studies regarding DOHaD, the concept of intrauterine programming of diseases was proposed and improved.

Moisiadis and Matthews studied the role of endogenous glucocorticoids (GCs) (cortisol in humans and corticosterone in rodents) in fetal programming in 2014, believing that maternal GC overexposure may be the main cause of fetal programming and susceptibility to diseases in offspring [14]. Studies conducted in our laboratory also found that PEE increased fetal exposure to maternal GCs by stimulating the maternal hypothalamus-pituitary-adrenal (HPA) axis and opening the placental GC barrier, increasing susceptibility to a variety of conditions such as MS, non-alcoholic fatty liver disease (NAFLD), and depression in adults [2, 15]. It was further found that the expression level of insulin-like growth factor 1 (IGF1) in the fetal liver determined the birth weight, organ structure, and functional development of the fetus [16–18]. Furthermore, PEE affected the level of GCs to regulate the expression of IGF1 [19, 20]. A cohort from Weeks et al. also reported that adults (22.2 to 44.4 years old) with fetal alcohol spectrum disorders (FASDs) caused by PEE had an increased incidence of metabolic abnormalities, including type 2 diabetes, low HDL, high triglycerides, and female-specific overweight and obesity [21]. Therefore, changes in intrauterine neuroendocrine metabolic programming may be the key mechanism for a variety of neuropsychiatric and metabolic diseases in IUGR offspring caused by PEE [22]. However, there is no systematic experiment to support this claim or hypothesis to explain it.

Currently, research on the developmental toxicity of PEE has made significant progress, but there is a lack of full understanding of its characteristics on offspring impacts, susceptibility to multiple diseases in utero programming, and mechanisms of disease occurrence in PEE-induced adulthood. According to the work of our laboratory and that of other researchers, this review outlines the characteristics of offspring developmental toxicity caused by PEE, the role of neuroendocrine programming and epigenetics in it, sex differences and possible mechanisms, suggesting that alterations in the HPA axis and GC-IGF1 axis programming may be the core mechanisms for the susceptibility to metabolic-related diseases in offspring caused by PEE, and proposing “dual programing” in utero and the “two strikes” theory of disease occurrence in adulthood caused by PEE. We wish to promote understanding of the toxicity caused by PEE and the mechanisms involved, to provide new perspectives on the early prevention and treatment of ethanol developmental toxicity-related fetal-derived diseases.

## Short- and long-term effects of PEE

Many products contain ethanol, such as all baijiu, wine products, some skin-care products, dyes, paint removers, gasoline, disinfectants, detergents, and pesticide residue on food. Pregnant women often consciously or unconsciously contact the above items. Among these, maternal drinking has the most obvious damage. In this section, according to different occurrence times of effects on the fetus, the developmental toxicity of ethanol is divided into short-term toxicity and long-term toxicity. Adverse effects on embryo and fetus are defined as “short-term toxicity,“ and, on postnatal offspring, they are called “long-term toxicity.“

Drinking alcohol during development of the embryo or fetus may cause miscarriage. The more drinks that are consumed and the longer the drinking occurs, the greater the possibility of spontaneous abortion. Drinking more than three days a week was associated with a higher risk of miscarriage [23–27]. IUGR is defined as the limitation on the growth and development of embryos or fetuses, which is characterized by multiple organ dysfunction, growth retardation, and low birth weight [22, 28]. IUGR is also one type of short-term toxicity caused by PEE [29–32]. An epidemiological investigation showed that the birth weights of offspring from mothers who drank were significantly lower than those of the control group [33]. Mouse studies found that acute ethanol exposure that occurred twice during pregnancy might inhibited cell proliferation during the trimester one, cell migration and differentiation during trimester two, and cellular communication and neurotransmission during trimester three in offspring [34]. Studies in our laboratory also suggested that ethanol exposure during middle and late pregnancy significantly reduced the birth weight in mice, and the IUGR rate was as high as 80% [35]; moreover, ethanol exposure led to abnormal development or function in many types of tissues and organs in the fetus, such as the hippocampus, hypothalamus, adrenal gland, cartilage and bone (Table 1) [36–39].

In addition to short-term toxicity, PEE can also lead to long-term toxicity (Table 1). “Catch-up growth” is defined as the phenomenon by which longitudinal growth velocity transiently stands above the statistical limits of normality for age and/or maturity after the removal of a growth-inhibiting condition, which has been shown to be associated with increased susceptibility to various conditions including osteoarthritis, MS, insulin resistance and other metabolic diseases such as diabetes and obesity [37–41]. PEE induced low birth weights, while mounting epidemiological and experimental studies have shown that low-birth-weight individuals frequently present IGF1-induced “catch-up growth” after birth [37, 40], causing physical and mental abnormalities and increased susceptibility to MS and various metabolic diseases in adulthood [42, 43]. Studies showed that PEE increased apoptosis and inhibited proliferation of islet β cells and decreased insulin secretion in rat (at 13 weeks of age) and guinea pig (on postnatal day 150–200) before and after birth [8, 44, 45]. Our studies also showed that the adult offspring of rats exposed to ethanol during pregnancy had increased blood glucose levels when fed a normal diet and elevated blood glucose levels, insulin levels, and insulin resistance index when fed a high-fat diet, leading to decreased glucose tolerance and diabetes at postnatal week 17 and 24 [2, 41]. The mechanism of these effects is related to abnormal development of the pancreas mediated by programming alteration of the GC-IGF1 axis [46]. In rats, offspring with IUGR induced by PEE also showed “catch-up growth” in body weight, elevated blood triglyceride levels, increased liver lipid and glycogen synthesis, and inhibited lipid output, and these rats were more likely to have typical NAFLD phenotypes than the control rats [2]. In addition, we found that PEE of rats caused retardation of bone growth, inhibition of endochondral ossification, delayed development of primary and secondary ossification centers [36], and poor cartilage quality in offspring before and after birth [39]; these findings were consistent with those of Simpson et al. [47]. Moreover, the persistent poor cartilage quality before and after birth (at postnatal week 24) caused by PEE in rats was related to the low functional programming of local IGF1 signaling, while high-fat diet after birth increased blood cholesterol content and deposition in cartilage, which further induced osteoarthritis [38].

**Table 1:** Short- and long-term adverse effects on offspring of PEE

Species	Time	Dose	Tissues/organs	Short-term adverse effects	Long-term adverse effects	Reference
**Humans**						
	First trimester	1–2 drinks/day*	–	Elevated spontaneous abortion	–	[23]
	Gestational week 6–16	5 units^@^ or more alcohol/week	–	Elevated spontaneous abortion	–	[24]
	–	10 or more drinks /week^#^	–	Elevated 2–3 (females)/2–5 (males) times spontaneous abortion	–	[25]
	Gestational week 0–20	4 + drinks/week	–	Elevated miscarriage	–	[26]
	Gestational week 0–10	4 + drinks/week	–	Miscarriage is strongest for miscarriage occurring prior to 10 weeks of gestation; women who drank only spirits had more than a two-fold increased risk of miscarriage	–	[27]
	Gestational week 28–36	100 g/week	–	Poor fetal growth is increased; IUGR	–	[29]
	Throughout pregnancy	Three drinks or more	–	Decreased birthweight	–	[30]
	First trimester	Four drinks/week	–	Reduction in fetal growth	–	[31]
	Gestational months 1 and 7	Low-to-moderate drinking	–	IUGR, preterm delivery, low birthweight	–	[32]
	Gestational weeks 20–44	1–2, 3–4, and ≥ 5 drinks/week	–	SGA, interaction between prenatal alcohol consumption and smoking	–	[33]
**Mice**						
	GD8–11, 14–16	5 g/kg	Brain	–	Disrupted cell proliferation, migration differentiation and communication and neurotransmission (PD4-60)	[34]
	GD11–17	6.4 g/kg·d	HPA axis	IUGR, inhibition of fetal HPA axis activity	–	[35]
**Wistar rats**						
	GD9–20	4 g/kg·d	Bone	Suppressed osteoclast differentiation	–	[36]
	GD9–20	4 g/kg·d		Adrenal developmental abnormality	CORT level was decreased; partial “catch-up growth” (PW4 and 24)	[37]
	GD11–20	4 g/kg·d	Cartilage	Chondrodysplasia	“Catch-up growth”; Susceptibility to osteoarthritis (PW4 and 24)	[38, 39]
	GD11–20	4 g/kg·d	Liver	Low body weight; hyperglycemia; hepatocyte ultrastructural changes	“Catch-up growth”; increased the susceptibility to NAFLD (PW1, 4 and 24)	[2]
	GD 11 until term delivery	4 g/kg·d	Hypothalamus/pituitary gland/liver	-	“Catch-up growth”; enhanced susceptibility to MS; neuroendocrine metabolic programming (PW16 and 20)	[41]
	GD9–20	4 g/kg·d	Blood/pancreas	Serum glucose and insulin levels as well as pancreatic β cell mass were reduced	Pancreatic dysplasia and impaired insulin biosynthesis (PW12 and 24)	[46]
	GD9–20	4 g/kg·d	Testis	Morphological abnormality; low serum testosterone	Testicular dysplasia; low serum testosterone levels (PW6 and 12)	[48]
	GD9–20	4 g/kg·d	Ovary	Decreased number and proliferation of oocytes, and increased apoptosis of oocytes	Increased atretic follicles; susceptibility to premature ovarian insufficiency; decreased serum estradiol (E2) levels (PW6 and 12)	[49]
**Sprague Dawley rats**						
	Throughout pregnancy	4 g/kg·d	Muscle/pancreas	Decreased body size; inducing insulin resistance and beta-cell dysfunction	“Catch-up growth”; impairs glucose tolerance (at 4. 7 and 13 weeks of age)	[8]
	Throughout 21 days of gestation	15%, 25%, or 36% ethanol-derived calories	Skeleton	Decreased fetal body weight, length, and skeletal ossification; growth retardation; restricted bone development; increased offspring risk of osteoporosis later in life	–	[47]
	GD11-20	35% of daily calories from ethanol	–	–	Enhanced ethanol intake and the behavioral response to ethanol odor in adult (PD30-90)	[50]
**Guinea pigs**						
	Throughout gestation	4 g/kg·d	Prefrontal cortex/liver	–	Increased liver weight; metabolic dysregulation; neurobehavioral teratogenicity (PD150-200)	[44]
	Throughout gestation	4 g/kg·d	Adiposity/ pancreas	Growth restriction at birth	“Catch-up growth”; increased visceral and subcutaneous adiposity; reduced insulin production and/or secretion(PD100-140 and 150–200)	[45]
	GD2–67	4 g/kg·d	Hippocampus	–	Behavioral and cognitive deficits (PD60)	[51]
**Sheep**						
	GD95-133	0.75 g/kg	Kidneys	Reduction in nephron endowment	–	[52]
	GD9-135	0.75 g/kg	Lung	Surfactant phospholipid concentration was reduced and the composition was altered by ethanol exposure	The adverse effects of ethanol exposure on lung do not persist to 2 month after birth	[53]

* In calculating total alcohol consumption, one and a half glasses of wine were considered equivalent to a glass of beer or measure of spirits (“one drink”);@ The author divided the number of alcoholic drinks into no alcohol, 1–4 units and 5 + units per week according to the investigation; # one drink = 12 g of alcohol; IUGR, intrauterine growth retardation; SGA, small for gestational age; E, embryonic day; GD, gestational day; PD, postanal day; PW, postanal week; NAFLD, non-alcoholic fatty liver disease; MS, metabolic syndrome; HPA, hypothalamus-pituitary-adrenal; CORT, corticosteron

## Characteristics of developmental toxicity induced by PEE

Epidemiological investigations showed that fetuses suffered from FAS when pregnant women were exposed to ethanol at 3.0–4.3 g/kg·d [54], or even at doses as low as 0.35 g/kg·d [55]. Simpson et al. found that when pregnant rats were fed diets of equal calories but different ethanol proportions, the fetal offspring in the high-dose group (36% ethanol-derived calories) showed significant reductions in body weight and length and inhibition of ossification, while only a delay in radius was observed in the low-dose group (15% ethanol-derived calories) [47]. It has been suggested that the higher the ethanol dose during pregnancy, the more developmental toxicity to the fetus. However, there are reported differences in the toxicity of low-dose ethanol exposure to the fetus. The results of the epidemiological investigations of Kelly et al. and Robinson et al. indicated that the toxicity of light alcohol (1–2 drinks per week or per occasion in Kelly’s study; 2–6 standard drinks per week in Robinson’s study) consumption during pregnancy was minimal or could not be observed in the offspring [56, 57]. However, Gray et al. believed that these negative results have arisen from, for example, the lack of rigorous experimental design, short observation times, and incomplete detection indicators. They suggested that due to the different metabolic rates of ethanol among individuals, peak blood alcohol levels are higher and residual time is longer in pregnant women with slow metabolism; therefore, low-dose ethanol consumption can seriously impair fetuses in this subpopulation [58]. The findings of Lewis et al. corroborate those of Gray et al. in a study of 15,000 pregnant women, Lewis et al. found that despite the same amount of ethanol consumed during pregnancy, the intelligence quotient (IQ) scores of the 8-year-old children of women with ethanol metabolic enzyme deficiency were lower than those of women with normal ethanol metabolic enzyme levels [9]. Moreover, Lewis et al. also suggested that the genetic variation of both mother and fetus is an important moderator of the fetal effects of alcohol [9]. This suggests that the fetal toxicity induced by PEE is not only related to the amount of ethanol consumed but also to the ethanol metabolic capacity and genetic variation of the mother and fetus. Therefore, Gray indicated that the determination of a ‘safe’ level which can be uniformly recommended for all pregnant women seems unrealistic [58].

Time is another important factor in the toxicity induced by PEE. Short-term exposure to high doses of ethanol directly damages fetal tissue, especially during stages of fetal development, resulting in miscarriage, stillbirth, and malformation [59, 60]. Fish et al. found that after acute exposure to 2.8 g/kg ethanol twice in early pregnancy, the morphology of the cerebellum, hippocampus, striatum, and corpus callosum changed in fetal mice. In the offspring of male mice (on postnatal day 28–45), activity increased in maze tests and the preference for a rich environment decreased, and in the female offspring, exploratory behavior increased and cage running decreased [61]. Diaz et al. also found that acute ethanol exposure in mid-pregnancy (12 or 15 days of pregnancy) was associated with neurobehavioral abnormalities in offspring of early and late adolescent or young adult Sprague Dawley rats, but differed with exposure time, species (although there are few reports), and sex [62]. Typically, these abnormalities were greater in Long Evans males and Sprague Dawley female rats [62]. Chronic ethanol exposure often causes persistent damage in the middle and late stages of pregnancy and leads to serious fetal developmental toxicity (such as IUGR [35]). A study by Dettmer et al. showed that chronic ethanol exposure during pregnancy caused long-term inhibitory effects on N-methyl-d-aspartate receptor subtype 2B (NR2B) (a glutamate-gated ion channel) in the cerebral cortex of guinea pig offspring and upregulated the expression of glutamate receptor subunits (GluR2/3); these changes may be related to the neurobehavioral changes observed in guinea pig offspring on postnatal day 61 [63]. A study by Iqbal et al. showed that PEE led to changes in the protein level of the γ-aminobutyric acid type A (GABA-A) receptor β-2/3 subunit in the adult offspring of guinea pigs and damaged their spatial learning ability, which was potentially related to FAS and long-term harm [51]. Previous studies in our laboratory found that ethanol exposure during middle and late pregnancy in rats caused the dysfunction of glucose lipid metabolism in liver and HPA axis, therefore increasing the susceptibility of adult offspring to NAFLD, MS, and cholesterol-accumulation-related osteoarthritis, and there were differences between males and females [2, 35, 38, 39, 41, 64].

In summary, PEE, whether acute or chronic exposure (at least at high doses), causes a variety of adverse effects in offspring. Ethanol exposure in early pregnancy leads to structural abnormalities of the nervous system and adverse pregnancy outcomes, while in middle and late pregnancy, it is more likely to lead to IUGR, functional abnormalities, and susceptibility to multiple diseases in adulthood.

## Developmental toxicity induced by the direct action of ethanol

In adults, 90–98% of absorbed ethanol is metabolized in the liver, while only 2–10% is directly excreted from the kidneys and exhaled from the lungs. Ethanol and its main metabolite, acetaldehyde, are small hydrophilic compounds that can directly enter the fetus through the placental barrier. After drinking three to five bottles of alcoholic drinks (approximately 150 mg/dl), the average human blood alcohol concentration is 33 mM, and the blood ethanol concentration of alcoholics is approximately 20–170 mM [65]. Previous work in our lab indicated that at a maternal ethanol consumption of 4 g/kg·d in rats, the serum ethanol concentration in the mother and fetus was 87 mM and 58 mM, respectively [2], indicating that ethanol can easily cross the placenta into the fetus. Cumming et al. indicated that the activity of liver alcohol dehydrogenase (ADH) in a fetus during late pregnancy or in newborn sheep was only 7% that of adults, and ADH activity in the placenta was even lower [66]. Pikkarainen and Räihä measured the activity of ADH in humans, and found that although fetal ADH activity in the second month of pregnancy was detectable, it was just 3–4% of the adult level. With an increase in gestational age, the activity of ADH gradually increases, but is always lower than that of adults [67]. Because the capacity of the fetal liver to metabolize ethanol is not mature, the actual metabolic burden in the developing fetus may be much higher than that in adults under similar conditions. In other words, the fetus is more prone to the direct toxic effects of ethanol. Chen et al. found that ethanol directly inhibited the Wnt/β-catenin pathway through oxidative stress to delay the differentiation of bone mesenchymal stem cells (BMSCs) and bone dysplasia, or directly upregulated IGF1 signaling pathway to cause ovarian cell apoptosis and premature ovarian insufficiency in female Sprague-Dawley rats [68]. Sun et al. also found that PEE can affect individual metabolism of endogenous and exogenous substances in early adulthood due to the direct effect of ethanol on impairment of protein levels and enzyme activities of cytochrome P450s (CYPs) in rats [69]. These results indicate that ethanol causes direct toxicity to multiple fetal tissues and organs *via* many mechanisms.

## Developmental toxicity mediated by fetal exposure to excessive maternal glucocorticoids induced by PEE

In most previous studies, the developmental toxicity of ethanol was believed to be related to the direct action of ethanol on a fetus. However, in recent years, the influence of elevated levels of maternal-derived GCs induced by PEE has attracted more and more attention. Although current studies have shown that PEE increases maternal and fetal blood ethanol levels in a dose-dependent manner [2, 35, 70–72], there has been no direct comparison of the effects of different doses of prenatal ethanol on fetal serum GC levels in humans. The reports are different in animal models, but the existing literature suggests that the higher the dose of prenatal ethanol and the longer the duration, the higher the maternal and fetal GC levels [2, 35, 70–72].

### Maternal glucocorticoid overexposure in offspring induced by PEE

The placenta is crucial for normal fetal development. Placental 11β-hydroxysteroid dehydrogenase type 2 (11β-HSD2) and P-glycoprotein (P-gp) are key mediators of maternal GC inactivation and transport [73–76]. 11β-HSD2, the key enzyme catalyzing GC oxidation, is believed to be the “gate-keeper” of the placental barrier against GCs [77, 78]. Because adrenal gland function is immature during the fetal period, fetal GC levels mainly originate from the mother through the placental GC barrier [79, 80]. Population and rodent studies showed that placental 11β-HSD2 activity was easily affected by various adverse conditions during pregnancy, resulting in exposure of the developing fetus to excessive maternal GCs [81, 82]. The concentration of cortisol in the serum and urine of drinkers was significantly higher than that in non-drinkers [83–86]. We also confirmed in rats that PEE increased maternal GC levels *via* a stress response and opened the placental GC barrier (ethanol directly downregulated 11β-HSD2 expression through cAMP/PKA/EGR1 signaling), resulting in fetal exposure to excessive maternal-derived GCs and inducing IUGR [35].

Placental ATP binding cassette (ABC) transporters protect placental and fetal tissues by excreting exogenous and endogenous metabolites. ABCB1/MDR1, namely P-gp, is the most abundant drug efflux transporter expressed in the membrane on the maternal side of the placental syncytiotrophoblast, representing another GC barrier on the placenta. P-gp transports GCs back to the maternal side against the concentration gradient, which limits the entry of GCs into placental cells and fetuses, thus reducing fetal exposure to maternal GCs [87]. Studies have suggested that the expression of P-gp decreased in the intestinal epidermal cells of mice exposed to ethanol [88]. Our previous study also showed that the expression of P-gp in rat placenta decreased after exposure to ethanol alone [89], the mechanism of which is related to expression and activity changes in the JNK/YB-1/P300 pathway [90]. In addition, P-gp inducers can decrease maternal-derived GCs and IUGRs of offspring caused by adverse environmental conditions (including ethanol exposure) during pregnancy [90]. It is suggested that the expression and functional inhibition of placental P-gp are also involved in the opening of the placental GC barrier caused by PEE. In conclusion, changes in the placental barrier caused by PEE caused fetal overexposure to maternal-derived GCs.

### Excessive exposure of fetuses to maternal glucocorticoids mediates offspring IUGR and postnatal susceptibility to multiple conditions

Basal levels of GCs play an important role in regulating the biosynthesis and metabolism of saccharides, fat, and protein and the proliferation and differentiation of cells. GCs are not only important factors in regulating fetal development and maturity, but they are also critical in determining the fate of the fetus after birth. Maternal GCs are the main source of fetal GC levels [35, 80]. A large number of reports suggest that high maternal GCs program the susceptibility of offspring to multiple conditions after birth [22, 79]. Human offspring with IUGR were found to have increased cortisol concentrations of umbilical cord blood at birth [91]. A variety of adverse factors during pregnancy can lead to increased fetal GCs and abnormal fetal development [81, 82, 92, 93]. Our previous studies also confirmed that the blood corticosterone levels of IUGR fetal rats exposed to ethanol during pregnancy were significantly higher than those of the control group; under these conditions, activity of the HPA axis in the maternal rats increased, while the synthesis of fetal adrenal steroids was inhibited, suggesting that maternal-derived GC overexposure occurred in the fetal rats [2, 35]. Furthermore, the increased expression of fetal liver triglyceride (TG) synthase, decreased neuronal activity in the paraventricular nucleus (PVN) region of the fetal hypothalamus, decreased activity of the fetal adrenal IGF1 signaling pathway, and inhibition of osteoclast differentiation at the osteo-cartilage interface were found to be mainly related to high maternal GCs induced by PEE [2, 35–37]. These studies all indicate that overexposure to maternal GCs may be another important mechanism of offspring IUGR and the susceptibility to multiple diseases in adulthood caused by PEE.

## Mechanism of maternal-derived glucocorticoid-mediate intrauterine neuroendocrine metabolic programming induced by PEE

Intrauterine programming refers to the process of permanent changes in morphology and function of embryonic or fetal tissues and organs [22]. Under physiological conditions, a variety of neuroendocrine hormones are involved in programming the development and function of multiple fetal organs. As a core component of the “DOHaD”, intrauterine programming is also responsible for pathologies (susceptibility to multiple conditions). However, to date, the intrauterine programming mechanism of IUGR in offspring has not been systematically clarified. Recently, a series of studies by us and other researchers may have confirmed that alterations in intrauterine neuroendocrine metabolic programming induced by maternal GC overexposure were associated with susceptibility to multiple chronic diseases in adults [22, 94, 95].

### Intrauterine maternal glucocorticoid overexposure and developmental programming of the HPA axis

The HPA axis, a neuroendocrine axis, plays an important role in prenatal and postnatal stress and defense responses. Mounting studies from human or animals have indicated that PEE causes changes in an offspring’s HPA axis, which are manifested as low basal activities from the intrauterine to early postnatal periods and high stress sensitivity throughout life [14, 96–98].

#### Low basal activity programming of the HPA axis

Although conclusions were not entirely consistent, accumulating studies have shown that the HPA axis has low basal activity in IUGR rats exposed prenatally to ethanol [96–99]. Previous studies from our group also found that the blood adrenocorticotropic hormone (ACTH) and corticosterone levels in IUGR rats induced by PEE were lower after birth than those of the control rats [41, 64]; it was further confirmed that fetal rats exposed to alcohol during pregnancy had low adrenal function, mainly manifested by reduced expression of steroidogenic acute regulatory protein (StAR) and cytochrome P450 family 11 subfamily A member 1 (P450scc) [35, 37], suggesting that the low basal activity of the HPA axis caused by PEE originated from the fetus.

The concentration of basal GCs during the fetal period and normal development of the fetal adrenal gland determine fetal maturity and postnatal fate [14, 100, 101]. It was found that development of adrenal function in IUGR offspring was delayed, resulting in low-activity programming of the HPA axis, which is consistent with the low activity of the HPA axis observed in offspring exposed prenatally to ethanol [97]. Our previous studies found that ethanol exposure during middle and late pregnancy inhibited the functional development of the HPA axis in fetal rats, which was manifested through fetal overexposure to maternal GCs, decreased corticotrophin-releasing hormone (CRH) and arginine vasopressin (AVP) levels in the hypothalamus, and decreased StAR and P450scc (the key genes of adrenal steroid hormone synthesis) expression [35, 37, 102]. Moreover, fetal maternal GC overexposure inhibited the steroid synthesis function of fetal adrenal cortical cells in a concentration-dependent manner, the mechanism of which was mainly related to the hypofunctional programming of adrenal corticosteroid synthesis induced by the GC activation system, including 11β-HSD1/2, GC receptor (GR), and CCAAT/enhancer binding protein α (C/EBPα). These effects continued after birth and even into adulthood [37]. Therefore, the changes in functional programming of the adrenal gland caused by maternal-derived GC overexposure induced by prenatal ethanol are an important reason for the intrauterine origin of the low basal activity of the HPA axis.

#### Stress hypersensitivity programming of the HPA axis

A number of studies from humans and animals have confirmed that stress hypersensitivity of the HPA axis is one of the key mechanisms in MS (e.g., diabetes and NAFLD) and mental disorders (e.g., depression and schizophrenia) susceptibility originating from fetuses [97, 103–106]. PEE programmably alters the stress sensitivity of the HPA axis in human and animal offspring [35, 97], but its specific mechanism has not been fully clarified. Our previous studies found that PEE in rats reduced serum ACTH and CORT levels by 65% and 60%, respectively, but there were no significant differences between PEE and control groups after chronic stress. Moreover, the increased rates of ACTH and CORT concentrations in offspring after PEE were significantly higher than those in offspring without exposure [41], suggesting that the offspring exposed prenatally to ethanol had stress hypersensitivity of the HPA axis, which was consistent with other basic research and clinical reports [97, 98, 107, 108]. Furthermore, we found that, in the offspring of rats exposed to ethanol during pregnancy, excitatory potential increased in the PVN of the hypothalamus whether examining fetal or adult offspring and this effect was characterized by invariable expression of an excitatory glutamate transporter (vesicular glutamate transporter 2 (VGluT2)) and decreased expression of inhibitory GABA synthase (65 kDa glutamic acid decarboxylase (GAD65)), resulting in a significant increase in the VGluT2/GAD65 ratio [109]. It is suggested that PEE permanently changes the setting point and sensitivity of hypothalamic PVN in offspring, resulting in stress hypersensitivity of the HPA axis.

As an advanced regulatory center, the hippocampus plays an important role in the HPA axis. The affinity of hippocampal mineralocorticoid receptor (MR) for GC is more than 10 times that of the GR [110]. When the GC level is low, almost all the GCs bind to the MR to regulate basal activity of the HPA axis; when GC levels increase, saturating the MR, GC then binds to the GR, resulting in glutamate release [111] and attenuation of the overactive HPA axis to the basal state through the Glu-GABA synaptic connection [112, 113]. Therefore, the balance of hippocampal MR/GR expression is critical to regulate HPA-axis activity, which directly determines the ratio of hippocampal VGluT2/GAD65 [104, 114]. It has been found that hippocampal GR participates in perinatal programming of the HPA axis by negatively regulating the expression of CRH [115]. After birth, the balance of MR and GR is maintained [116, 117]. However, once they are imbalanced, dysfunction of the HPA axis occurs, which is a typical response of the hippocampus to chronic stress or depression. Our studies found that PEE increased GR expression in fetal rats, leading to decreased MR/GR and increased VGluT2/GAD65 expression ratios. When the offspring of female rats exposed prenatally to ethanol were subjected to chronic stress (cool water swimming), the expression of GR in the hippocampus was higher than that of the control rats, the expression ratio of MR/GR was further reduced, and the VGluT2/GAD65 ratio was further increased. At the same time, pathological changes such as cell arrangement disorder, deep nuclear staining and shrinkage in the dentate gyrus (DG) and CA3 areas of the hippocampus were aggravated [102, 109]. These results suggest that PEE reduces negative regulatory effects on the hypothalamus to enhance its excitatory potential. The above changes can continue into adulthood. After chronic stress, the hippocampus damage is aggravated and the regulatory ability to HPA axis is further reduced, leading to hyperexcitation of the hypothalamus and finally to stress hypersensitivity of the HPA axis.

### Intrauterine maternal glucocorticoid overexposure and developmental programming of the GC-IGF1 axis

IGF1 and its downstream signals are involved in regulating the development, differentiation, and metabolism of tissues and organs during the intrauterine period [16, 18, 118]. IGF1 regulates cell proliferation and apoptosis, and glucose and lipid metabolism by binding to the IGF1 receptor (IGF1R) [119]. Although IGF1 is expressed in almost all embryonic tissues [17], it is mainly produced in the liver during the fetal period. Hepatic IGF1 or IGF1R knockout or mutation significantly reduces fetal birth weight and length [120–122]. Studies confirmed that blood IGF1 levels decreased in fetuses with IUGR in mammals, and these levels were significantly higher in IUGR offspring exhibiting “catch-up growth” than in those not exhibiting “catch-up growth” [123–125], which is related to adult metabolic diseases [2, 126]. A study in our laboratory also showed that the expression of liver and serum IGF1 in fetuses of rats exposed to ethanol during pregnancy was lower than that of controls [2], while they also increased significantly after birth, and the increased rate was higher than that of the controls, which may be one of the intrauterine programming mechanisms of NAFLD [2].

**Fig. 1:**
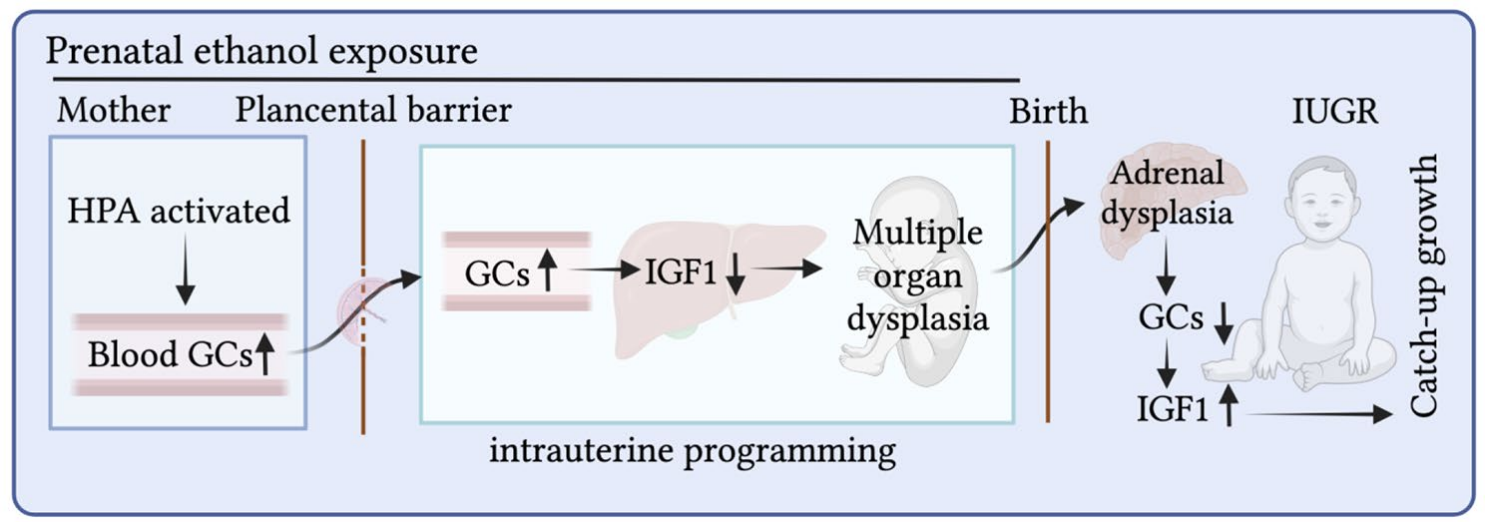
GC-IGF1 axis mediates programming changes in the offspring exposed prenatally to ethanol. HPA, hypothalamic-pituitary-adrenal; GCs, glucocorticoids; IGF1, insulin like growth factor 1; IUGR, intrauterine growth retardation

GCs promote fetal maturation, while this process is theoretically accompanied by growth inhibition. Increasing numbers of studies have shown that high levels of GCs inhibit the expression of IGF1 in various tissues and cells of human and sheep [19, 20]. A series of studies confirmed that PEE increases the level of serum corticosterone in fetal rats, but decreases the levels of serum and liver IGF1. After birth, the levels of corticosterone decrease, while those of IGF1 increase. After a high-fat diet, corticosterone levels in the offspring further decrease, but IGF1 levels further increase and body weight shows “catch-up growth” [2]. Similarly, when fetal blood corticosterone levels in male IUGR offspring rats exposed to ethanol prenatally increase, the expression of IGF1 and downstream steroid synthase enzymes in the adrenal gland decreases. After birth, when corticosterone levels decrease in the offspring of rats, the expression of adrenal IGF1 and downstream steroid synthase enzymes shows a compensatory increase [37]. This negative change suggests that there may be an axial relationship between GC and IGF1 in multiple tissues (such as the liver and adrenal gland) (Fig. 1), which may be a physiological axis of fetal development and maturity, mediating adaptive changes and compensatory effects under the adverse intrauterine environment.

To confirm how maternal GC overexposure mediates programming alterations of the GC-IGF1 axis in fetuses, researchers systematically observed changes in corticosterone levels, the adrenal GC activation system, IGF1 signaling, and steroid synthesis before and after birth in IUGR rats induced by PEE in a rat model [37]. The results showed that maternal-derived GCs increased the expression of 11β-HSD1; decreased the expression of 11β-HSD2; increased the expression of MR, GR, and C/EBPα; and inhibited C/EBPβ and IGF1 signaling in fetal adrenal glands. Moreover, endogenous corticosterone content and the expression of corticosteroid synthase system decreased in the offspring exposed prenatally to ethanol. In the early postnatal period, these offspring showed low basal activity of the HPA axis and suppression of the adrenal GC activation system, but enhanced IGF1 signaling; after a postnatal high-fat diet, the HPA axis showed stress hypersensitivity, accompanied by local GC-IGF1 axis-mediated changes in organs and tissues (such as the adrenal gland and liver) [2, 64, 127]. This suggests that GC-IGF1-axis programming plays an important role in maintaining activity of the HPA axis and regulating organ and tissue functions before and after birth.

## Epigenetic modifications in intrauterine programing and genetic toxicity induced by PEE

Epigenetics refers to heritable regulation of gene expression that does not involve gene sequence changes and is a reflection of environmental stimuli on genetic factors. Examples include DNA methylation, histone modification, and non-coding RNAs [128, 129]. It is believed that epigenetic changes are involved in the intrauterine origin of adult-onset diseases and are responsible for the continuation of fetal programming that is induced by adverse intrauterine environments into the postnatal period and even the next generation of offspring [79]. Studies have suggested that PEE induces epigenetic changes in various organs and cells (Table 2) [130, 131]. As early as the 1990s, Garro et al. found that acute ethanol (3 g/kg twice a day) exposure from the ninth to the eleventh day of gestation caused extensive DNA hypomethylation in fetal mouse [132]. Recently, News et al. reported that pregnant mouse exposure to alcohol (2 g/kg) on embryonic 18.5 could result in incorporation of alcohol into gestating fetal brains and contribute to rapid histone acetylation in the brain in part by direct deposition of alcohol-derived acetyl groups onto histones in an ACSS2-dependent manner [133]. Based on the whole embryo culture technique, Liu et al. also found that alcohol exposure (88 mM) during mouse embryonic neurodevelopment induced alterations in the DNA methylation of the fetal brain, mainly occurring on chromosomes 7, 10, and X, which further caused delayed closure of the neural tube [134].

In addition, PEE in mice can alter acetylation of key genes and the expression of non-coding RNAs (lncRNA1354, miR-467b-5p, and miR-302c) in the hippocampus, liver and sperm cells of offspring to affect the development of tissues and organs in adults (postnatal day 87 or 70) [135, 136]. Moisiadis and Matthews reviewed multiple publications and found that excessive maternal GCs in fetuses can induce universal DNA methylation, histone acetylation, and miRNA expression, which might participate in fetal programing and intergenerational inheritance of fetal-derived neurological, cardiovascular and metabolic diseases [79]. Moreover, studies from our group indicated that PEE decreased H3K9 and H3K14 histone acetylation within the IGF1 promoter and increased IGF1 gene expression in multiple fetal organs of rats (e.g., the liver and bones), triggering altered GC-IGF1 axis programming (high GC and low IGF1 in intrauterine; low GC and high IGF1 after birth); these epigenetic changes were inherited by the F2 generation [127]. Stouder et al. also found that PEE in mice caused hypomethylation of the imprinted gene H19 in the somatic and spermatogonial cells of offspring, which was transferred to F2 generation [137]. These epigenetic changes in imprinted genes and the GC-IGF1 axis partly explain the intrauterine origin, long-term effect and multigenerational inheritance of developmental toxicity in offspring induced by PEE [136]. PEE can also lead to expression and promoter methylation changes in common imprinted genes such as IGF2 [44, 138, 139]. However, it mediates developmental toxicity and multigenerational inheritance effects in PEE offspring and whether there are other mechanisms involved in PEE-induced multigenerational inheritance needs to be further studied.

**Table 2:** Effects of PEE on epigenetic modifications of genes in multiple tissues

Species	Periods	Dose	Tissue	Genes	Epigenetics	Outcomes	Generations	Reference
**Human**								
	20 to 70.5 weeks postconception	–	Brain	–	Decreases in 5mC, H3K4me3, H3K9ac, H3K27ac, H4K12ac, and H4K16ac	Stillbirth	F1	[140]
	gestation	205 ± 32.8 g/day	Cerebellum	TET1↓, GABRD↓	DNA hypermethylation	Regulating cerebellar pathophysiology	F1	[141]
**Long-Evans Rats**								
	GD1–GD22	3.5 or 4.5 g/kg	Hippocampus	Dnmt1↑, Dnmt3a↑, MeCP2↑	DNA hypermethylation	Teratogenesis in the hippocampus	F1	[130, 142]
**Wistar Rats**								
	GD9–GD20	4.0 g/kg	Liver	IGF1 P450scc	Low H3K9ac	Metabolic abnormalities	F1, F2	[127]
			Adrenal glands			Adrenal corticosterone synthesis dysfunction	F1, F2	[143]
**Mice**								
	GD9–GD11	3.0 g/kg	Individual fetus	Throughout the genome	DNA hypomethylation	Developmental abnormalities	F1	[132]
	GD1.5–2.5/6.5	2.9 g/kg	Embryos and placentae	H19/ CTCF1	DNA hypomethylation	Growth retardation	F1	[144]
	GD10–GD18	0.5 g/kg	Tail/liver/tibialis anterior muscle/ sperm cell	H19	DNA hypomethylation	Decreased spermatogenesis	F1, F2	[137]
	GD8.25	88 mM	Embryo	Nlgn3, Elavl2, Sox21, Sim1	Hypomethylation	Abnormal fetal development	F1	[134]
				Cyp4f13	Hypermethylation			
	GD12.5 for 5 days in vitro	13, 26, 70 mM	Neurosphere stem cell	Throughout the genome	H3K4/H3K27 Hypermethylation	Developmental abnormalities	F1	[145]
					H3K9ac			
				lncRNA1354↓	ncRNA			
	GD0–GD8.5	10% for 2 ml	Hippocampus	VGluT2↑, miR-467b-5p↓	Hypomethylation ncRNA	Hippocampal dysfunction	F1	[135]
	GD14–16/GD16	2.5 g/kg	Brain	miR-302c↑	ncRNA	FAS	F1	[136]
	GD18.5	2.0 g/kg	Hippocampus	Fstl1, Cep152, Uimc1 and so on	Histone acetylation	-	F0, F1	[133]

GD, gestational day; GABRD, δ subunit GABAA receptor; Dnmt1, DNA methyltransferase 1; Dnmt3a, DNA methyltransferase 3α; MeCP2, methyl-CpG binding protein 2; H19, H19 imprinted maternally expressed transcript; Nlgn3, neuroligin-3; Elavl2, ELAV-like RNA binding protein 2; Sim1, SIM BHLH transcription factor 1; Cyp4f13, cytochrome P450 family 4 subfamily f polypeptide 13; VGluT2, vesicular glutamate transporter 2; IGF1, insulin like growth factor 1; P450scc, cytochrome P450 family 11 subfamily A member 1; FAS, fetal alcohol syndrome

## “Dual programing” and “two strikes” mechanisms mediate multiple fetal-originated Diseases caused by PEE

“Thrifty phenotypes” are fetal adaptive changes, along with nutrition and energy redistribution, which maintain the normal development and function of crucial organs when fetuses were exposed to adverse environments during pregnancy. However, such individuals become susceptible to diseases in adulthood [146]. Thus, we hold that when a mother is exposed to ethanol during pregnancy, on the one hand, ethanol directly enters the circulation of the fetus to injure fetal tissues; on the other hand, under conditions of high maternal GCs, the expression of 11β-HSD2 and P-gp decreases, and the placental GC barrier opens. Then, the maternal-derived GCs injure fetal tissues and modulate IGF1 signaling in various fetal organs and tissues to trigger the redistribution of nutrition and energy to protect the development of crucial organs (such as liver and brain), while attenuating the development of non-critical organs (such as bone and cartilage) to maintain fetal survival. This makes the fetus “thrifty.“

After birth, with the withdrawal of maternal-derived GCs and low GC expression in the offspring themselves, liver IGF1 expression continues to increase [37]. Therefore, the levels of liver and serum IGF1 in the offspring exposed prenatally to ethanol were significantly higher than those in the control group for a long time after birth, along with notable postnatal “catch-up growth” [2], but the blood levels of growth hormone (GH) were consistently lower (data unpublished). In other words, the excessive increase in hepatic IGF1 expression mediated by GCs is responsible for the “catch-up growth” of IUGR offspring, rather than the traditional GH-IGF1 axis. Interestingly, although GC-IGF1 axis programming (high GC and low IGF1 in intrauterine; low GC and high IGF1 after birth) was altered in IUGR offspring subjected to PEE, the change in expression of genes involving liver glycolipid metabolism was not altered. Based on these findings, we have proposed that this process be defined as the “one programing” where the functional changes in various organs are caused by GCs under PEE and continue to adulthood. Meanwhile, the programming changes in multiple organ functions mediated by the GC-IGF1 axis were defined as “another programing,“ which mainly reflects the pre- and postnatal adaptive and compensatory changes in the multi-organ functions of offspring exposed to ethanol during pregnancy.

The HPA axis showed low basal activity and hypersensitivity to chronic stress in IUGR offspring induced by PEE, as well as the GC-dependent phenotype of glucose and lipid metabolism [41, 64]. Those findings indicated that the excessive maternal-derived GCs induced by PEE and ethanol itself caused the “first strike” to the fetuses, which disrupted fetal development of organs and further induced adaptive alterations. Outside of the adverse intrauterine environment and under conditions of adequate postnatal nutrition, the offspring demonstrated “catch-up growth” through redistribution of nutrients and energy to compensate for the organ dysplasia. However, it was confirmed by the rat and zebrafish embryo models that those changes might disturb fetal-originated metabolic programing and exacerbate the abnormal morphology of tissues and organs and dysfunction of glucose and lipid metabolism to exhaust the metabolic potential of the offspring, thus increasing susceptibility to metabolic diseases [2, 126, 147, 148]. When the offspring again suffered from adverse factors postnatally, such as high-fat diet or chronic stress [41] (these were defined as the “second strike”), various metabolic diseases developed. Therefore, “dual programing” (including the “one programing” and “another programing”) and “two strikes” (including the “first strike” and “second strike”) induced by PEE mediate the dysplasia of multiple organs and susceptibility to various diseases in the offspring.

## Sex-based differences and mechanism of developmental toxicity induced by PEE

Although the developmental toxicity of ethanol has been reported in both female and male animals and the high sensitivity of the HPA axis is very consistent between sexes, numerous studies still suggest that there are sex-based differences in severity and specific manifestations [61, 62].

### Sex-based differences caused by placental barrier

Studies have shown that PEE inhibits the expression and activity of 11β-HSD2 and P-gp more significantly in female placentas than males [149]. The suppressed expression and activity of 11β-HSD2 are related to the higher 11β-HSD2 DNA methylation level in female rat placenta [150]. When Sprague Dawley rats were exposed to ethanol in the middle and late stages of pregnancy, the expression of 11β-HSD2 was decreased in female placenta and increased in male placenta [149]. The difference in PEE between male and female placentas makes it easier for maternal-derived GCs to enter female fetuses, resulting in sex-based differences in developmental toxicity. Furthermore, high maternal cortisol levels have been reported to induce GC resistance in male fetuses, while female fetuses remain sensitive [151]. In addition, the GR gene is mainly composed of five subtypes: GRα, GRβ, GRγ, GRA, and GRP [152, 153]. GRα has the strongest activation effect on target genes; GRA, GRβ, and GRP cannot bind to GCs [154, 155]; GRP is related to GC resistance; and GRγ only has ~ 50% of the activation effect of GRα [156]. Human placental trophoblasts express 12 GR protein subtypes, which makes the placenta uniquely sensitive to GCs [157]. Under the high intrauterine GC levels induced by PEE, the male placenta develops GC resistance by increasing GRβ, GRA, and GRP expression, while the female placenta increases GC sensitivity by decreasing expression of GRβ and enhancing the interaction between GRαA, GRαD3, and GRαC [157]. Therefore, the differences in placental barrier and GC sensitivity are the reasons for the sex differences in developmental toxicity caused by PEE. However, whether the difference in GC sensitivity of other tissues and organs mediates the sex difference in developmental toxicity of PEE needs to be further studied.

### Sex-based differences caused by neuroendocrine metabolic programming

The differences in HPA axis development and sensitivity might also be responsible for the sex differences in susceptibility to adult-onset diseases [158]. Studies from humans have demonstrated that the basal activity and sensitivity of the HPA axis are much higher in females than in males [159, 160]. In rats, as the basal serum corticosterone levels, corticosterone secretion rate, and reactivities to ACTH and corticosterone in female are higher than those in males in physiology, the HPA axis is also activated more easily in females than in males, especially during proestrus when serum estrogen reaches its peak, which further increases the activity and sensitivity of the HPA axis [161, 162]. Our previous studies observed that PEE increased blood corticosterone concentration in rat offspring, upregulated GR expression in the hippocampus, decreased or did not change the expression of IGF1 and GAD67 from the intrauterine to postnatal phase in females, but increased the expression of GR, IGF1, and GAD67 in the male offspring hippocampus prenatally and in adulthood [102, 109]. Additional studies from both mice and rats indicated that PEE disrupted development of the hypothalamic-pituitary-gonadal (HPG) axis and delayed its maturation [163, 164], which may also be one of the reasons for sex differences. PEE caused postponed adolescence, abnormal vaginal development, abnormal estrogen synthesis and secretion, and sexual behavior changes in female animals [1, 165–167]; however, abnormal testicular development (including a decreased number of interstitial glands), vesicles in the seminiferous tubules, suppressed testosterone synthesis and secretion, hyposensitivity to luteinizing hormone (LH), feminization, hyposensitivity of the HPG axis and sexual debility were observed in the male offspring of rats [7, 168–172].

Furthermore, the HPG axis and HPA axis can mutually regulate each other’s functions at various tissue levels [97]. For instance, estradiol can promote ACTH release during stress, thereby increasing GC levels, while progesterone competes with estradiol to inhibit HPA axis activity; only when progesterone is at extremely low levels does the regulatory effect of estrogen on the HPA axis become evident [161, 162]. Testosterone can inhibit CRH mRNA levels and reduce the body’s responsiveness to stress [162, 173]. Moreover, the CRH gene promoter region contains estrogen and androgen response elements, and estrogen can promote CRH transcription in the hypothalamus, while androgens inhibit CRH transcription; therefore, testosterone can reduce corticosterone production, while estrogen has the opposite effect [97, 162, 173]. This may be one of the reasons why females are more sensitive to stress compared to males and why the basal activity and sensitivity of the HPA axis in female offspring induced by PEE are higher than in males [159, 160]. In addition, an increase in GC levels inhibited the activity of 17β-hydroxysteroid dehydrogenase (an important enzyme in testosterone synthesis) in the testes, leading to a reduction in serum testosterone levels in males [174], which can also inhibit the release of gonadotropin-releasing hormone (GnRH) from the hypothalamus and interfere with the release of follicle-stimulating hormone (FSH) and LH induced by GnRH, thereby reducing estrogen levels [175]. IGF1 is also important for the development of the HPG axis, including testis and ovaries [175–177]. Studies in our lab also found that PEE caused elevated blood corticosterone levels and decreased expression of IGF1 in the liver, testes and ovaries of the fetal rats [2, 37, 49], which resulted in ovarian and testicular dysplasia, therefore decreasing estrogen and testosterone levels in female and male offspring, respectively [48, 49]. In summary, all HPA, HPG and GC-IGF1 axes may be involved in the sex difference of developmental toxicity caused by PEE.

## Summary and prospects

**Fig. 2:**
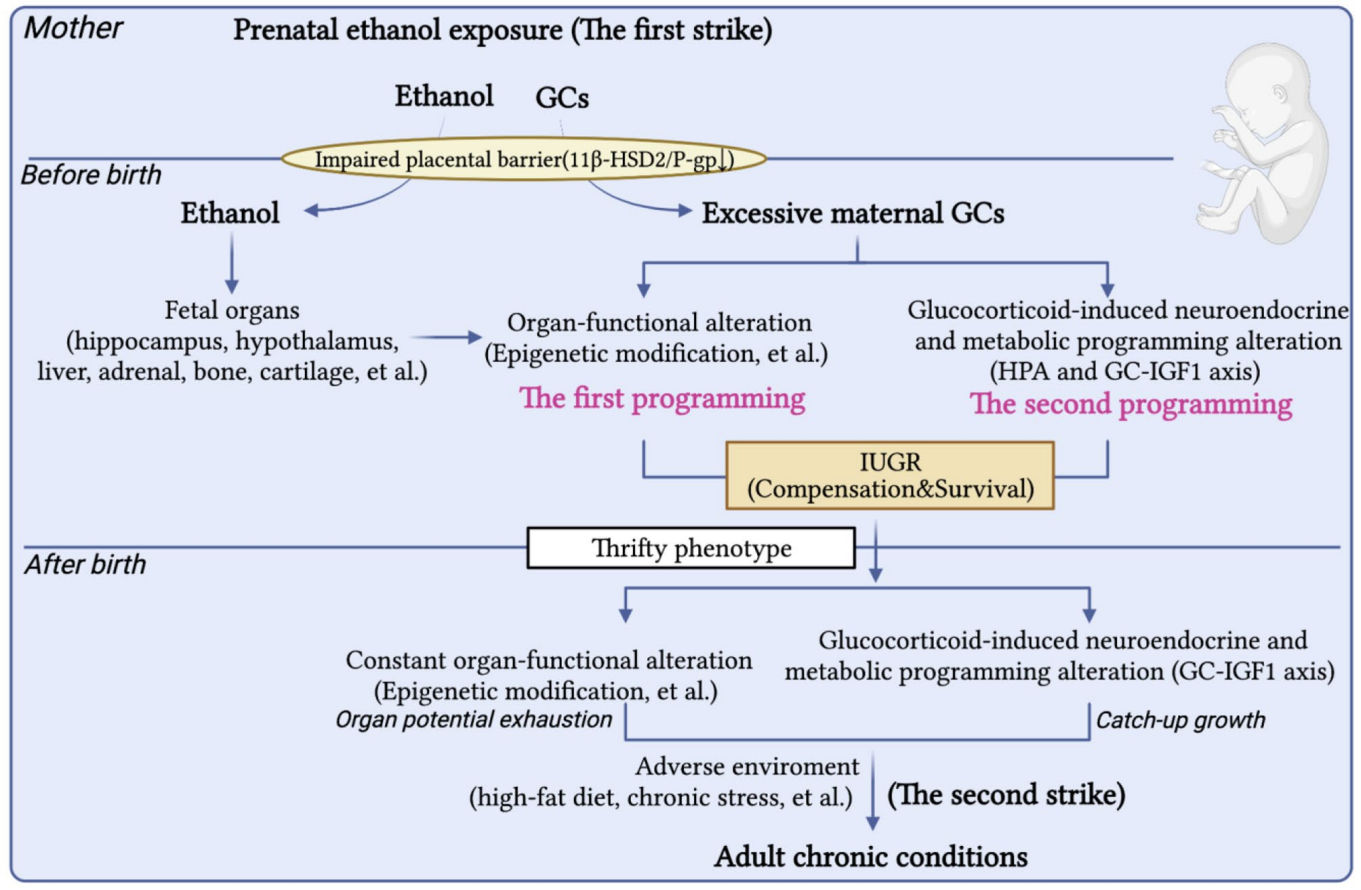
The intrauterine programing mechanism of adult metabolic diseases induced by PEE. GC, glucocorticoid; IGF1, insulin like growth factor 1; IUGR, intrauterine growth retardation; 11β-HSD2, 11β-hydroxysteroid dehydrogenase 2; P-gp, P-glycoprotein; HPA axis, hypothalamic-pituitary-adrenal axis

The mechanism of dysplasia of fetal tissues and organs and susceptibility to fetal-originated diseases in offspring caused by PEE is not only related to the direct actions of ethanol or its metabolites, but also related to the fetal neuroendocrine metabolic programming changes (HPA and GC-IGF1 axis programming). The “dual programing” and “two strikes” mechanisms induced by the direct effects of ethanol and neuroendocrine programming lead to the occurrence of various metabolic diseases in adult offspring (Fig. 2). Epigenetic modifications, placental barrier and neuroendocrine metabolic programming and HPG axis participated in sex-based differences and intergenerational inheritance induced by PEE, but some key points of these are still absolutely unclear.

Additionally, there are other common neuroendocrine axes (systems) with important roles in the human including the hypothalamic pituitary thyroid axis (HPT) and renin angiotensin system (RAS) except for HPA, GC-IGF1, and HPG axes. Hannigan and Cudd et al. showed that PEE led to a decrease in triiodothyronine (T3) and T4 levels in Long-Evans rats and goat offspring, both prenatally and after birth, while having no significant effect on free T4 [178, 179]. Wilcoxon et al. also indicated that PEE could result in permanent programming changes in the HPT axis in offspring rats and behavioral and cognitive dysfunction in offspring rats, and decreased expression of GAP-43 and GR in hippocampus, while supplementing with T4 can reverse these changes [180, 181]. Moreover, studies by Fidalgo and us also suggested that PEE inhibited the binding of angiotensin (Ang) IV to Ang II type 1α receptor (AT1R), increased the expression of angiotensin converting enzyme (ACE), Ang II, and AT1R in the serum and kidney, while decreasing the expression of ACE2, AT2R, and Mas receptor (MasR), thereby eliminating the memory consolidation effect of exogenous Ang IV on male offspring aged 3–6 months, and causing kidney and bone dysplasia, as well as adult (24 weeks after birth) nephrotic syndrome and osteoporosis [182–184]. All of these indicate that PEE may also lead to changes in other neuroendocrine axes such as HPT and RAS. However, current related studies are still limited, mostly at the level of animals and observational studies, lacking in-depth programming mechanisms and epidemiological investigations. There have also been no reports on whether maternal glucocorticoid overexposure caused by PEE is involved in changes in the HPT axis and RAS system. At the same time, due to differences in exposure time and dose, there are still many inconsistencies in the existing literature [52]. Therefore, more systematic research is urgently needed.

Finally, with accumulating research on fetal-originated diseases, translational medicine is continuing to bring basic research to clinical practice or clinical applications. Though avoiding ethanol exposure during pregnancy could be the best option, in cases where exposure has already occurred, based on the temporal characteristics of ethanol-induced developmental toxicity, sex differences, epigenetic programming and “dual programing” and “two strikes” mechanisms, interventions of ethanol exposure during early pregnancy primarily should focus on fetal structural abnormalities and abnormal pregnancy outcomes, while ethanol exposure during mid to late pregnancy primarily should aim to prevent long-term effects and increased susceptibility to multiple diseases in adult; moreover, specific interventions can be targeted towards male or female offspring based on the reasons for sex differences, respectively, while epigenetics may serve as diagnostic and warning markers or intervention targets; meanwhile, voiding a second strike after birth is also one of the effective strategies to prevent fetal-originated diseases caused by PEE. However, due to the some unclear molecular mechanism of the developmental toxicity and susceptibility to various diseases caused by PEE, the established targets at present are greatly limited. Therefore, based on neuroendocrine metabolic programming, the primary and secondary prevention of birth defects induced by prenatal adverse environments (e.g., ethanol exposure) needs to be further studied.

## Data Availability

All data are included in the manuscript.

## Abbreviations

PEE: Prenatal ethanol exposure
IUGR: Intrauterine growth retardation
FAS: Fetal alcohol syndrome
MS: Metabolic syndrome
DOHaD: Developmental Origins of Health and Disease
HPA: Hypothalamus-pituitary-adrenal
NAFLD: Non-alcoholic fatty liver disease
IGF1: Insulin-like growth factor 1
FASDs: Fetal alcohol spectrum disorders
GC: Glucocorticoid
SGA: Small for gestational age
GD: Gestational day
PD: Postanal day
PW: Postanal week
CYP: Cytochrome P450
CORT: Corticosteron
IQ: Intelligence quotient
NR2B: N-methyl-d-aspartate receptor subtype 2B
GluR2/3: Glutamate receptor subunits 2/3
GABA-A: γ-aminobutyric acid type A
ADH: Alcohol dehydrogenase
BMSCs: Bone mesenchymal stem cells
11β-HSD2: 11β-hydroxysteroid dehydrogenase type 2
P-gp: P-glycoprotein
ABC: ATP binding cassette
TG: Liver triglyceride
PVN: Paraventricular nucleus
ACTH: Adrenocorticotropic hormone
StAR: Steroidogenic acute regulatory protein
P450scc: P450 family 11 subfamily A member 1
CRH: Corticotrophin-releasing hormone
AVP: Arginine vasopressin
GR: Glucocorticoid receptor
C/EBPα: CCAAT/enhancer binding proteinα
VGluT2: Vesicular glutamate transporter 2
GAD65: 65 kDa glutamic acid decarboxylase
MR: Mineralocorticoid receptor
DG: Dentate gyrus
IGF1R: Insulin-like growth factor 1 receptor
GABRD: δsubunit GABAA receptor
Dnmt1: DNA methyltransferase 1
Dnmt3a: DNA methyltransferase 3α
MeCP2: Methyl-CpG binding protein 2
H19: H19 imprinted maternally expressed transcript
Nlgn3: Neuroligin-3
Elavl2: ELAV-like RNA binding protein 2
Sim1: SIM BHLH transcription factor 1
Cyp4f13: Cytochrome P450 family 4 subfamily f polypeptide 13
GH: Growth hormone
HPG: Hypothalamic-pituitary-gonadal
GnRH: Gonadotropin-releasing hormone
FSH: Follicle-stimulating hormone
HPT: Hypothalamic pituitary thyroid axis
RAS: Renin angiotensin system
T3: triiodothyronine
T4: Thyroxine
Ang: Angiotensin
AT1R: Angiotensin II type 1α receptor
ACE: Angiotensin converting enzyme
MasR: Mas receptor
